# Smell and Taste Alterations in Patients Receiving Curative or Palliative Chemotherapy—The CONKO 021—ChemTox Trial

**DOI:** 10.3390/cancers16142495

**Published:** 2024-07-09

**Authors:** Tobias Bleumer, Janine Abel, Wolfgang Böhmerle, Sebastian Schröder, Soo Ann Yap, Nigel Dross Engelbert Schaeper, Thomas Hummel, Sebastian Stintzing, Lars Uwe Stephan, Uwe Pelzer

**Affiliations:** 1 Department of Hematology, Oncology and Tumor Immunology, Berlin Institute of Health, Charité-Universitätsmedizin Berlin, Freie Universität Berlin, Humboldt-Universität zu Berlin, 10117 Berlin, Germany; tobias.bleumer@charite.de (T.B.); janine.abel@charite.de (J.A.); sebastian.schroeder@charite.de (S.S.); soo-ann.yap@charite.de (S.A.Y.); nigel.schaeper@charite.de (N.D.E.S.); sebastian.stintzing@charite.de (S.S.); lars-uwe.stephan@charite.de (L.U.S.); 2 Department of Neurology and Experimental Neurology, Berlin Institute of Health, Charité-Universitätsmedizin Berlin, Freie Universität Berlin, Humboldt-Universität zu Berlin, 10117 Berlin, Germany; wolfgang.boehmerle@charite.de; 3 Smell & Taste Clinic, Department of Otorhinolaryngology, Technische Universität Dresden, 01307 Dresden, Germany; 4 Department of Internal Medicine, Bundeswehrkrankenhaus Berlin, 10115 Berlin, Germany

**Keywords:** chemotherapy, taste and smell alterations, dysosmia, dysgeusia, chemosensory function, neuropathy, CIPN, anorexia

## Abstract

**Simple Summary:**

Taste and smell alterations (TSAs) are a distressing yet underdiagnosed side effect in cancer patients undergoing chemotherapy. However, long-term investigations using both questionnaires and chemosensory tests are scarce. We examined the prevalence of quantitative and qualitative TSAs, as well as their connection with clinical characteristics such as age, sex, anorexia, and neuropathy. We also compared patients receiving perpetual or temporary chemotherapy. All patients were examined up to five times within 12 to 24 months, commencing before the beginning of chemotherapy. We found TSAs in approximately 4 out of 5 patients during chemotherapy. The highest prevalence was documented among patients above 60 years of age as well as among those reporting anorexia or presenting signs of neuropathy. Post-therapy, taste and smell function recovered; however, scores did not reach baseline levels within 6 to 12 months. Future investigations will assess potential interventions to prevent or reduce TSAs.

**Abstract:**

Previous data regarding chemotherapy-induced olfactory and gustatory dysfunction (CIOGD) are heterogeneous due to inconsistent study designs and small numbers of patients. To provide consistent, reliable data, we conducted a cohort study using standardized testing. Patients diagnosed with lymphoma, leukemia, or gastrointestinal malignancies were examined up to five times (T1 to T5), beginning prior to chemotherapy. We examined patients receiving temporary treatment up to 12 months post-therapy. Clinical assessment included extensive questionnaires, psychophysical tests of olfactory and gustatory function, and measurement of peripheral neuropathy. Statistical analysis included non-parametric tests to evaluate the longitudinal development of CIOGD. Our data (n = 108) showed a significant decline in olfactory and gustatory testing during chemotherapy (*p*-values < 0.001). CIOGD appeared stronger among patients above 60 years, while sex did not matter significantly. However, we identified distinct associations between CIOGD and reported anorexia as well as with higher neuropathy scores. Self-assessment appeared less sensitive to chemosensory dysfunction than psychophysical testing. Post-therapy, olfactory and gustatory function regenerated, though baseline levels were not attained within 6 to 12 months. In conclusion, our data highlight the wide prevalence and slow recovery of CIOGD. Understanding CIOGD as a potential neurotoxic effect may disclose new therapeutic prospects.

## 1. Introduction

Global incidence of cancer is currently estimated at 20 million diagnoses per year [1]. Since the development and further advancement of chemotherapy in the 20th century, definite regimens have been used to treat a great variety of hematological and oncological diseases [2]. Hence, the number of patients relying on and receiving chemotherapeutics is expected to reach 15 million per year by 2040 [3]. One of the first curable malignancies using chemotherapy was Hodgkin’s lymphoma [4]. To this day, its standard treatment relies on chemotherapeutic substances and achieves long-term remission in over 90% of patients [5,6]. This is just one example among many types of cancer with dramatically increased survival rates and improved prognosis over the past 50 years [7]. Having this in mind, the long-term consequences of chemotherapy grow increasingly relevant. 

Chemotherapeutic agents target cells with high proliferation rates. Due to their inability to differentiate between malignant and physiological cells, patients frequently report or display various side effects. Nausea, alopecia, and fatigue having been among the most common for decades [8]. While chemotherapy regimens are updated and remission rates increase, long-term side effects like fatigue, negative prospects for the future, and taste and smell alterations become increasingly relevant [9,10,11]. Previous studies on the etiology of chemotherapy-induced olfactory and gustatory dysfunction (CIOGD) describe multiple determinants [12]. High proliferation rates of olfactory receptor neurons and gustatory sensory cells are the underlying pathophysiological factors [13]. Also, functional impairment through hyposalivation or xerostomia can add to chemosensory disorders [14]. Lack of concentration or memory disturbances can further modulate the correct sensation of smell and taste [15]. 

Consequently, CIOGD represents a frequently reported side effect in cancer patients. Current data range from 5% to 100%, with one systematic review suggesting a prevalence of 5 to 60% for dysosmia and 45 to 84% for dysgeusia [16,17]. However, results vary due to inconsistent study designs and often small numbers of patients [18,19]. Thus, longitudinal trials using standardized questionnaires and testing are needed to identify and help patients suffering from CIOGD [20]. Indisputably, CIOGD may reduce the quality of life and general condition of patients [21]. This is due to potential disturbances of food intake, suppressed appetite, and weight loss [13,22,23,24]. Also, CIOGD is associated with fatigue, nausea, and decreased food enjoyment [25,26,27]. Patients experiencing CIOGD even suffer social consequences as they share meals less often and show symptoms of depression more frequently [28,29]. Interestingly, recent data suggest potential connections between CIOGD and peripheral neuropathy. There have been multiple reports of patients with neuropathy also showing dysosmia or dysgeusia [30,31]. A recent study found hypogeusia in 59% of patients receiving taxane-containing chemotherapy [32]. To date, the extent and relevance of CIOGD-associated peripheral neuropathy remains indistinct. 

The aim of this study was to investigate olfactory and gustatory alterations in adult cancer patients receiving systemic chemotherapy using a longitudinal study design and standardized questionnaires as well as testing of olfaction and gustation. We evaluated associations with clinical characteristics such as age, sex, and anorexia, as well as with peripheral neuropathy. We assessed both quantitative and qualitative CIOGD during the course of our study as well as post-therapeutic recovery of olfactory and gustatory function.

## 2. Materials and Methods

This study was approved by the Charité—Universitätsmedizin Berlin ethical review committee (file reference: EA2/167/21) and was performed in accordance with the Declaration of Helsinki and the guidelines of Good Clinical Practice. Patients did not receive financial compensation for their participation.

### 2.1. Patients

This prospective, longitudinal, observational cohort study was conducted between July of 2021 and August of 2023. We determined the sample size using suspected gustatory and olfactory changes during chemotherapy. Pre-existing data showed mean threshold-identification scores of 21 points with a standard deviation of 5 points among healthy controls [33]. We suspected a decrease in olfactory performance to 18 points with a standard deviation of 10 points. The presumed power was set at 0.8, and the significance level was defined at α = 0.05. This resulted in an effect strength of 0.35 and a necessary sample size of 70 patients. Anticipating a drop-out rate of 35%, we planned to recruit 108 patients. 

Accordingly, we recruited 108 patients at the Department of Hematology, Oncology, and Tumor Immunology of Charité-Universitätsmedizin Berlin, Germany. Recruitment was performed prior to the first cycle of chemotherapy for either lymphoma, leukemia, or a gastrointestinal malignancy. Inclusion criteria for participation were (1) full capacity to consent to study participation; (2) physical condition at baseline of 0 or 1 regarding the ECOG-Score (Eastern Cooperative Oncology Group) [34]; (3) life expectancy of at least 12 months; (4) place of residence allowed anticipation of long-term participation; (5) age between 18 and 85 years; (6) able to read, speak, and understand German or English; (7) no previous or current symptomatic otorhinolaryngological comorbidities, including sinusitis and oral mucositis; (8) tobacco exposure of maximum 15 pack years; (9) no active COVID-19-infection or post-COVID-19-associated dysosmia or dysgeusia; and (10) no pre-existing diagnosis of neuropathy. Criteria for exclusion included (1) reduced capacity to consent; (2) physical condition at baseline of >1 regarding the ECOG-Score; (3) life expectancy < 12 months; (4) long-term treatment at our institution seems unlikely due to long distance to the patient’s place of residence; (5) age < 18 years or >85 years; (6) insufficient capability to communicate in German or English; (7) previous or current symptomatic otorhinolaryngological diagnoses; (8) tobacco exposure of >15 pack years; (9) current COVID-19-infection or post-COVID-19-associated dysosmia or dysgeusia; and (10) pre-existing diagnosis of neuropathy. 

### 2.2. Examination

**Figure 1:**
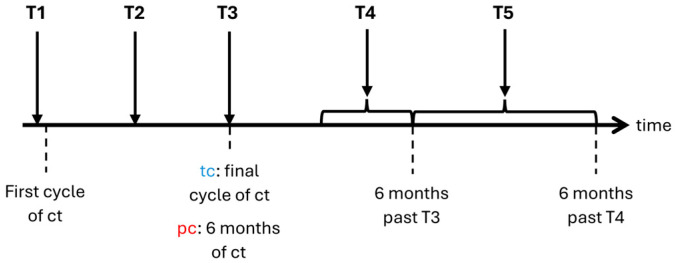
Timeline of examinations. ct: chemotherapy; tc: temporary chemotherapy; pc: perpetual chemotherapy; T1–T5: time points of examination.

Patients were screened prior to chemotherapy, which included taking a comprehensive medical history and performing a precise cancer classification within the TNM staging system before recruitment [35]. Participating patients were examined five times (T1 to T5) during a total time frame of 12 to 24 months. The first assessment (T1) was conducted prior to the first cycle of chemotherapy. Patients receiving temporary chemotherapy (i.e., curative intention) were examined a second time (T2) halfway through their regimen. Third assessment (T3) was taken at the end of the final cycle. We conducted the follow-up examinations 3 to 6 months (T4) as well as 6 to 12 months (T5) after completion of chemotherapy. No follow-up examinations were conducted in case of relapse because of chemotherapy re-challenge. Patients receiving perpetual chemotherapy (i.e., mostly with palliative intention) were assessed at 3 months (T2), 6 months (T3), 9 to 12 months (T4), and 12 to 18 months (T5) after baseline (Figure 1). We performed examinations during chemotherapy within the first 3 days of each particular cycle. Assessments during and after chemotherapy took place at the hospital or the outpatient department. 

Each examination consisted of a comprehensive questionnaire and a psychophysical examination of olfaction and gustation. First, we recorded sociodemographic parameters such as age, sex, body mass index, and profession. Second, we used the EORTC QLQ-C30 to assess patients’ health-related quality of life, as well as potential side effects of chemotherapy, such as reduced performance, lower energy levels, or changes in food intake [36]. Third, we used a standardized neurology-focused questionnaire extension to inquire about neurological symptoms, including signs of neuropathy like paresthesia or dysesthesia. Fourth, we recorded taste and smell alterations, as well as qualitative disturbances (parosmia, phantosmia, parageusia, and phantogeusia), and asked patients to self-assess their current olfactory and gustatory function. Answers were recorded using the web application Research Electronic Data Capture System (REDCap) [37,38]. 

Next, olfactory function was tested using the “Sniffin’ Sticks” [33]. These pen-like odor dispensers were used for a threshold and identification test. The “Sniffin’ Sticks” can also be used for the discrimination test, which was not performed to keep examinations within a feasible time frame of a maximum of 60 min. We performed threshold testing using 16 triplets of pens, with only one pen of each triplet actually containing an odor. We repeatedly presented the odor in increasing and decreasing intensity until the individual olfactory threshold was determined. The identification test uses another set of 16 pens with each pen containing a different odor of strong intensity. Patients were asked to identify the correct odor among 4 options. Both olfactory scores were added and resulted in the threshold-identification score (TI score). Published data were used to compare TI scores and categorize patients into groups of anosmia, hyposmia, normosmia, and supersmellers [39]. 

Subsequently, gustatory function was tested using the “Taste Strips” [40]. Blindfolded patients were presented with impregnated paper strips on their tongue and asked to identify sweet, salty, sour, and bitter flavors of different intensities [41]. Again, we used already published data to compare and categorize patients’ results into groups of hypogeusia and normogeusia. To date, gustatory testing using the “Taste Strips” does not offer a uniform definition of ageusia. 

We determined peripheral neuropathy at T1, T3, and T5 using nerve conduction measurement. Additional examination included testing of general esthesia, thermoception, pallesthesia, muscle strength, and reflexes. Results were quantified by the total neuropathy score-reduced (TNSr), with higher scores indicating more symptoms [42]. 

### 2.3. Data Analysis

We performed a descriptive analysis of sociodemographic parameters using means, median, standard deviation, frequencies, and percentages. The Friedman test was used to evaluate longitudinal data regarding the development of CIOGD (i.e., results of the olfactory and gustatory testing) and results of nerve conduction studies. We analyzed differences in sociodemographic and clinical factors between groups by means of Mann–Whitney tests and Kruskal–Wallis tests. In addition, we used Mann–Whitney tests and Kruskal–Wallis tests to evaluate differences in olfactory and gustatory tests as well as in nerve conduction studies between groups. Post-hoc analyses were used in case of significant results among pairwise comparisons. Using Spearman’s rank correlation, we evaluated correlations between self-assessments and psychophysical results. 

Subsets of patients were compared and evaluated using multiple subgrouping variables such as age categories, sex, and BMI categories. Age was categorized according to previous studies into groups of 18 to 40 years, 41 to 60 years, and >60 years [41]. The official BMI categories were adopted to categorize patients into groups of underweight, healthy weight range, overweight range, obesity range class I, obesity range class II, and obesity range class III [43]. In addition, diagnoses were grouped and compared with each other. Also, characteristics regarding chemotherapy regimens served as subgrouping variables. Important distinguishing features were known as neurotoxicity and temporary vs. perpetual therapy. Data analysis was performed using SPSS Statistics version 28 (Chicago, IL, USA). 

## 3. Results

### 3.1. Sociodemographic and Clinical Characteristics

The sociodemographic and clinical characteristics of all participating patients (n = 108) as well as of the two most important subsets—patients receiving temporary chemotherapy (i.e., most often following a curative intention of therapy; n = 58) and patients receiving perpetual chemotherapy (i.e., most often following a palliative intention of therapy; n = 50) are displayed in Table 1. Patients’ median age at baseline was 54.5 years, while patients receiving temporary chemotherapy (tc) were younger and had predominantly been diagnosed with lymphomas. Patients receiving perpetual chemotherapy (pc) suffered more often from gastrointestinal malignancies and showed higher scores of BMI at baseline. 

Over the course of our study, BMI decreased insignificantly to 24.4 kg/m^2^ at T4 and T5 among all patients. No differences were found between patients receiving tc and pc or other subsets of patients. However, the prevalence of anorexia showed a strong increase during chemotherapy from 21.3% at baseline to 65.9% at T2 and 80.5% at T3. From here, frequency diverged depending on the continuation of chemotherapy: in patients with tc frequency of reported anorexia dropped to 28.2% and 7.4% at T4 and T5, respectively. Among patients receiving pc, we documented anorexia in 70.8% and 88.2% of cases at T4 and T5, respectively. 

Throughout our study, a number of reasons led to increasing drop-out rates; 20 patients discontinued their participation due to changes in residence or continuation of treatment closer to their residence, 17 patients experienced relapse or needed therapy adjustments, 11 patients passed away, and another 16 patients were too stressed to continue participation (Table 2). Applied chemotherapeutics varied between treatment groups depending on the patient’s diagnosis (Table 3 and Table 4). 

### 3.2. Olfactory and Gustatory Self-Assessment 

At each examination, self-assessment of olfactory and gustatory function was obtained. At baseline, patients described their chemosensory function as “average” when compared to others. No significant differences were found between patients receiving tc and pc, between patients of different BMI categories, or between patients of different age groups. However, distinct differences showed between men and women (*p*-values for olfaction = 0.002; *p*-values for gustation = 0.053), with 30.6% of women describing their olfactory function as higher-than-average. The longer patients received chemotherapy, the worse their self-assessed functions became. However, olfactory function was still seen as “average” by the majority during chemotherapy. Overall, women and patients below 60 years of age evaluated their chemosensory function better than older patients did. Gustatory self-assessments showed similar dynamics; however, patients receiving tc described more decline during chemotherapy and showed stronger recovery after completion of chemotherapy. The percentage of patients with tc evaluating their taste sensation as “average” grew from 70% at T3 to over 80% at T4 and to over 90% at T5, while 42% and 30% of patients with pc described their taste sensation as moderately or severely below average at T4 and T5, respectively. Comparison of these results with chemosensory tests revealed lower sensitivity of self-assessment regarding both hyposmia and hypogeusia.

### 3.3. Olfactory Examination 

We conducted an olfactory examination using the “Sniffin’ Sticks”. At baseline, patients scored a mean of 8.1 points on the threshold test and a mean of 14.5 points on the identification test. On average, they scored 22.6 out of 32 possible points (SD 2.6). Patients prospectively receiving tc (23.0 points) scored higher than patients prospectively receiving pc (22.2 points; *p*-values = 0.01). Also, significant differences were found between patients above 60 years of age and patients between 18 and 40, as well as 41 to 60 years (*p*-values = 0.002 and *p*-values = 0.01, respectively). Likewise, women and underweight patients scored higher than men and patients within healthy weight or overweight ranges. Overall, men above 60 years of age reached the lowest scores of 21.0 points in total. 

Between baseline and T2, olfactory scores decreased from 22.6 to 18.7 points on average (*p*-values < 0.001). Again, men (17.7 points), patients above 60 years of age (16.8 points), overweight patients (18.2 points), patients reporting anorexia (17.9 points), and patients diagnosed with leukemia (18.1 points) reached lower scores. However, differences between temporary and perpetual chemotherapy (*p*-values = 0.71) as well as between neurotoxic and non-neurotoxic regimens did not reach significance (*p*-values among patients with tc = 0.71 and *p*-values among patients with pc = 0.25). 

Reduction of olfactory function continued between T2 and T3. Again, olfactory scores decreased significantly from 18.7 to 16.6 points (*p*-values < 0.001). No significant differences were shown between temporary and perpetual chemotherapy (*p*-values = 0.54). The same was true for neurotoxic therapies among patients with tc (*p*-values = 0.88). However, among patients receiving pc, those with neurotoxic therapies scored significantly lower than those without (*p*-values = 0.023). In men (16.0 points), patients above 60 years of age (15.7 points), overweight patients (16.0 points), patients reporting anorexia (16.2 points), and patients diagnosed with leukemia (16.3 points) reached lower scores. 

At T4, olfactory scores varied depending on the continuation of chemotherapy (*p*-values = 0.01). Patients who had finished tc showed olfactory recovery and scored 19.2 points on average. Among patients after tc, lower scores were reached by women (18.7 points), patients above 60 years of age (17.2 points), overweight patients (18.9 points), patients reporting anorexia (18.3 points), and patients diagnosed with gastrointestinal malignancies (17.7 points). In contrast, patients continuing pc showed further olfactory decline and scored 16.4 points at T4. Patients with neurotoxic therapy reached insignificantly lower olfactory levels than those without (*p*-values = 0.28). Again, men (15.2 points), patients above 60 years of age (15.0 points), overweight patients (15.1 points), patients reporting anorexia (15.7 points), and patients diagnosed with leukemia (15.6 points) reached lower scores.

This diverging trend continued at T5. Olfactory scores among patients after completion of tc increased to 20.3 points on average, while patients continuing pc scored 15.5 points (*p*-values < 0.001). Again, after tc, men scored higher than women, while women reached higher olfactory levels during pc. Other characteristics showed equivalent results as at T4. 

**Figure 2:**
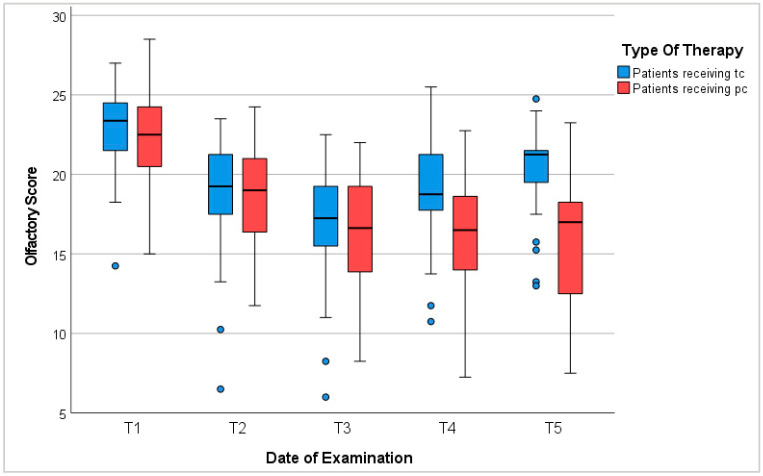
Olfactory score. T1: before chemotherapy; T2: halfway through tc or 3 months into pc; T3: at the end of tc or 6 months into pc; T4: 3–6 months after tc or 9–12 months into pc; T5: 6–12 months after tc or 12–18 months into pc; blue circles represent outliers.

In summary, the majority of patients suffered from olfactory dysfunction during treatment with chemotherapy. The degree and recovery of dysosmia varied depending on the duration of chemotherapy, sex, age, BMI, anorexia, cancer type, and chemotherapeutic neurotoxicity. In total, 90.2% of all patients experienced reduced olfaction during chemotherapy. In the case of tc, olfactory function recovered after completion of treatment. However, baseline levels were not reached within 6 to 12 months (Figure 2). 

### 3.4. Gustatory Examination

We conducted a gustatory examination using the “Taste Strips”. At baseline, patients scored a mean of 13.4 out of 16 possible points. (SD 2.6). Patients prospectively receiving tc scored 13.9 points, while those prospectively receiving pc scored 12.9 points (*p*-values < 0.001). Similar to olfactory results, overweight patients reached the lowest gustatory levels among all BMI categories (13.1 points). In addition, patients above 60 years of age scored significantly lower than younger patients. Men above 60 years scored the lowest, reaching 11.8 points on average. 

Between baseline and the second examination, gustatory scores decreased from 13.4 to 10.0 points on average (*p*-values < 0.001). Patients receiving tc scored insignificantly higher than patients receiving pc (*p*-values = 0.15). Similarly, no significant differences were observed between patients with and without neurotoxic chemotherapy regimens. We observed lower-than-average scores among men (9.1 points), patients above 60 years of age (8.5 points), overweight and obese patients (9.6 and 8.8 points, respectively), patients reporting anorexia (9.5 points), and patients diagnosed with leukemia and gastrointestinal malignancies (8.7 and 9.3 points, respectively). 

Patients’ gustatory function decreased further between T2 and T3, with average scores dropping from 10.0 to 8.6 points (*p*-values for patients receiving tc = 0.25; *p*-values for patients receiving pc = 0.06). Patients under perpetual treatment (7.1 points) scored significantly lower than patients at the end of temporary treatment (9.5 points; *p*-values = 0.01). No definite differences were shown between neurotoxic and non-neurotoxic chemotherapy regimens. Overall, lower gustatory scores were measured among men (7.6 points), patients above 60 years of age (6.5 points), and patients reporting anorexia (9.0 points). Results between BMI categories and diagnoses appeared ambiguous, with the exception of lymphoma patients who scored above average (10.4 points). 

Like olfactory results, gustatory scores diverged between T3 and T4 depending on the continuation of treatment (*p*-values between groups < 0.001). Patients still receiving chemotherapy showed a further decrease to 7.0 points on average. Among these, women (8.5 points) scored higher than men (5.7 points); patients between 18 and 40 years (10.6 points) scored higher than patients between 41 and 60 years (7.4 points), and patients above 60 years of age (5.2 points). Patients suffering from lymphomas (10.2 points) scored higher than those suffering from leukemia (7.4 points) or gastrointestinal malignancies (6.2 points). In addition, patients receiving neurotoxic chemotherapy scored 6.5 points, while those without scored 9.4 points (*p*-values = 0.1). In contrast, patients after completion of tc recovered to 11.4 points on average. Significant differences were observed between age groups (*p*-values for patients between 18 and 40 years compared to patients between 41 and 60 years = 0.02; *p*-values for patients between 18 and 40 years compared to patients above 60 years < 0.001) and patients reporting (12.0 points) and not reporting (9.9 points) anorexia (*p*-values = 0.04). 

Between T4 and T5, gustatory results continued diverging between therapy groups. Post-therapy patients recovered from 11.4 points at T4 to 12.5 points at T5. Among these patients, men’s scores (13.0 points) were higher than women’s scores (11.5 points), and patients reporting anorexia (8.3 points) scored lower than patients with intact appetite (12.8 points). On the other hand, patients receiving pc scored 7.7 points on average at T5. Significant differences were only seen between sexes, with women’s scores exceeding men’s scores (*p*-values < 0.001). Additionally, patients above 60 years of age (6.1 points), as well as patients diagnosed with leukemia (5.8 points) scored below average. 

**Figure 3:**
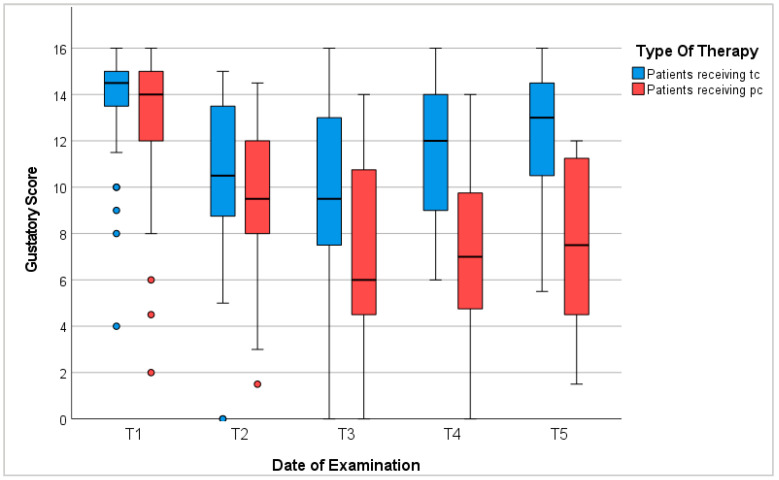
Gustatory score. T1: before chemotherapy; T2: halfway through tc or 3 months into pc; T3: at the end of tc or 6 months into pc; T4: 3–6 months after tc or 9–12 months into pc; T5: 6–12 months after tc or 12–18 months into pc; red/blue circles represent outliers.

In conclusion, 78% of patients in our study presented dysgeusia during receipt of chemotherapy. The degree of gustatory dysfunction depended mostly on the duration of therapy, sex, age, and anorexia. Gustatory recovery commenced post-therapy; however, patients did not reach baseline levels within 6 to 12 months (Figure 3). 

#### Sensation of Basic Tastes

Gustatory examination included specific testing of sweet, sour, salty, and bitter tastes. Similar to overall gustatory function, the sensation of each particular taste decreased during chemotherapy and showed recovery among patients receiving tc after completion of chemotherapy. Throughout the entire study, patients identified sweet taste most sensitively. On the opposite, we identified sour taste the least during chemotherapy. However, the detection of salty taste showed the slowest recovery after chemotherapy had ended. We measured the strongest recovery regarding bitter taste; however, none of the basic tastes recovered entirely within 6 to 12 months compared to baseline levels. 

**Figure 4:**
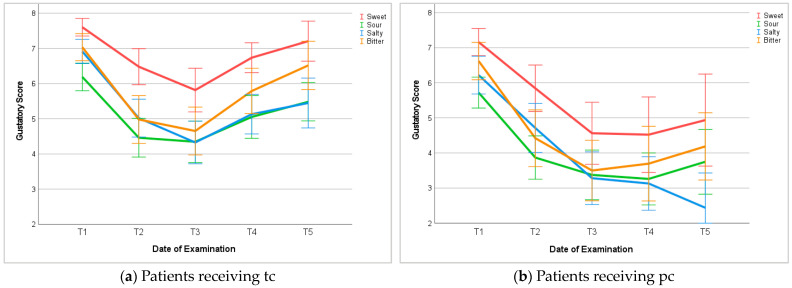
Gustatory examination of basic tastes. T1: before chemotherapy; T2: halfway through tc or 3 months into pc; T3: at the end of tc or 6 months into pc; T4: 3–6 months after tc or 9–12 months into pc; T5: 6–12 months after tc or 12–18 months into pc.

Among patients receiving pc, the perception of each particular basic taste decreased stronger than among patients receiving tc and showed slight to no recovery between T3 and T5. Again, throughout the course of examinations, patients correctly detected sweet taste more sensitively than other tastes. Between T3 and T5, patients’ ability to identify sweet, sour, and bitter tastes slightly improved. However, salty was least detected at T3, T4, and T5 and showed a continuous decrease compared to each previous examination (Figure 4). 

### 3.5. Qualitative Dysfunctions of Olfaction and Gustation

During receipt of chemotherapy, patients also showed qualitative dysfunctions of olfaction and gustation. The number of patients reporting parosmia grew to over 20% at T2, T3, and T4. Among patients who had finished tc, parosmia was no longer reported 6 to 12 months after treatment. This was also true for phantosmia, which was reported by less than 15% of all patients at any given time.

Compared to parosmia and phantosmia, qualitative gustatory dysfunctions were observed more frequently in our study. The number of patients reporting phantogeusia grew to 30% during temporary chemotherapy and 40% during perpetual chemotherapy. Post-therapy, patients recovered to baseline levels within 6 to 12 months. Moreover, parageusia appeared as the most frequently reported qualitative chemosensory dysfunction, peaking at 50% at the end of temporary treatment as well as after 12 to 18 months of perpetual treatment. Like the aforementioned qualitative dysfunctions, patients showed signs of recovery from parageusia within 6 to 12 months after completion of chemotherapy. 

### 3.6. Nerve Conduction Study 

**Figure 5:**
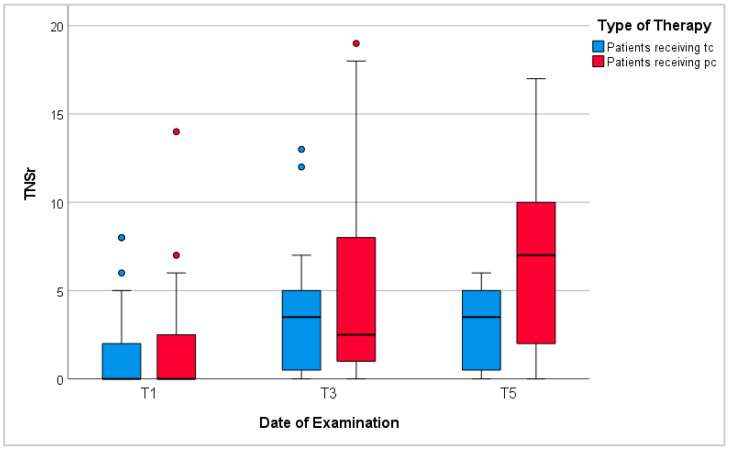
Total neuropathy score-reduced (TNSr). T1: before chemotherapy; T3: at the end of tc or 6 months into pc; T5: 6–12 months after tc or 12–18 months into pc; red/blue circles represent outliers.

Neurological examinations at T1, T3, and T5 showed an increase in symptoms of peripheral neuropathy during chemotherapy (Figure 5). Average TNSr tripled from 1.4 points at T1 to 4.2 points at T3 (*p*-values < 0.001). Patients receiving tc scored 3.3 points on average at T3. Among these, patients with neurotoxic treatment scored higher than those without (*p*-values = 0.07), while analyses of sex, BMI, diagnosis, and reported anorexia showed inconclusive results. Patients receiving pc scored 5.2 points on average at T3 (*p*-values compared to patients receiving tc = 0.14). Again, patients with neurotoxic treatment scored higher than patients without (*p*-values 0.03). Further analyses of sex, BMI, and anorexia showed no significant differences. Noticeably, patients with gastrointestinal malignancies scored significantly higher than patients with lymphomas (*p*-values = 0.002). Regarding all patients, those above 60 years of age scored significantly higher than younger patients. 

Between T3 and T5, TNSr rose from 4.2 points to 4.9 points on average with patients receiving pc scoring significantly higher than those having finished temporary chemotherapy (*p*-values 0.04). Similar to results at T3, analyses of BMI and reported anorexia showed no significant differences. However, men’s TNSr during receipt of pc were higher than women’s but recovered more quickly after completion of tc. In addition, during continued pc, patients with gastrointestinal malignancies showed significantly higher TNSr than patients with lymphomas (*p*-values = 0.002). Again, regarding all patients, those above 60 years of age showed more signs of peripheral neuropathy than younger patients. 

**Figure 6:**
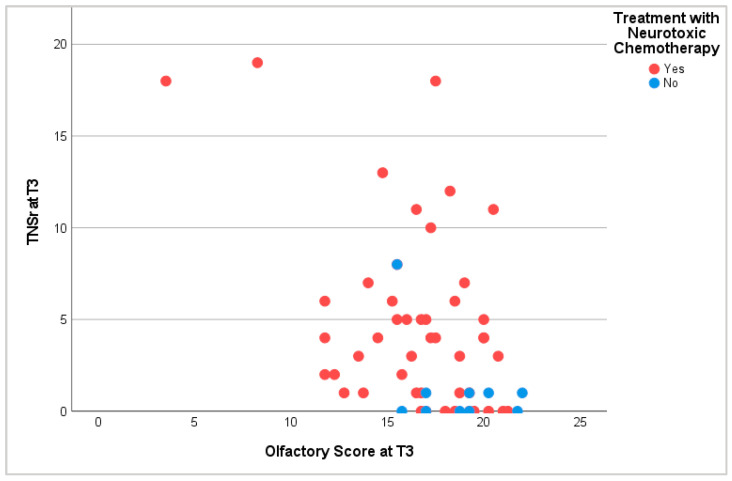
Olfactory score and TNSr at T3. T3: at the end of tc or 6 months into pc.

The comparison of neurological and chemosensory dysfunction revealed that patients without symptoms of peripheral neuropathy scored the highest points in olfactory and gustatory tests. Correspondingly, patients with dysosmia or dysgeusia showed symptoms of peripheral neuropathy more often. During and after treatment, patients with hyposmia and hypogeusia displayed higher TNSr than patients with regular chemosensory function. This proved especially true for patients receiving neurotoxic treatment (Figure 6). 

## 4. Discussion

Our data provide evidence to support the hypothesized correlation between the receipt of chemotherapy and dysfunctions of olfaction and gustation in adult cancer patients [17]. Current data based on both self-assessments and chemosensory testing are scarce [20]. This is one of few studies examining over 100 cancer patients for a minimum of 12 months in a longitudinal study design using standardized olfactory and gustatory tests as well as patient questionnaires. High rates of CIOGD were confirmed in our study, with approximately 4 out of 5 patients presenting TSA during chemotherapy. Additionally, to our knowledge, this is the first study to monitor peripheral neuropathy using neurological examination and nerve conduction studies to investigate potential correlations between chemosensory and neurological dysfunctions during receipt of chemotherapy. This potential correlation between dysosmia and peripheral neuropathy had previously emerged and was lately studied among patients receiving taxane-containing chemotherapy [31,32]. Here, 59% of patients reported dysgeusia, which was indeed correlated with symptoms of peripheral neuropathy. Our findings also suggest that cancer patients experiencing symptoms of peripheral neuropathy show gustatory and olfactory dysfunctions more often. This might indicate that chemosensory dysfunctions can be categorized as a neurotoxic side effect of chemotherapy that, in turn, could disclose new therapeutic approaches. 

Olfactory and gustatory functions are frequently impaired during and after chemotherapy. In our study, psychophysical testing appeared to be more sensitive in the detection of CIOGD than self-assessment. Similar results were found in previous studies using the same chemosensory tests [44]. However, other data also show contrasting results, with self-assessed CIOGD being more frequent [45]. Sensitivity of self-assessment appears to strongly depend on the style in which questions are phrased [46]. Furthermore, patients above 60 years of age were generally affected more strongly, while men showed more CIOGD during chemotherapy but recovered more quickly after treatment. These findings are mostly consistent with previous data showing higher rates of TSA among older patients [47,48,49]; however, some studies describe female sex as a potential risk factor for dysgeusia [50], while others do not mention or did not find sex as a relevant risk factor [20,51]. In addition, patients reporting anorexia showed higher rates of CIOGD. This correlation between anorexia and dysgeusia has also been described previously [24,52]. These gustatory dysfunctions can lead to decreased liking of food and reduced health-related quality of life [26,53]. In addition, patients diagnosed with gastrointestinal malignancies and those receiving neurotoxic treatment seemed to present CIOGD more often. Although we did not identify distinct correlations with particular types of cancer in our study, cancer progress must also be discussed as a separate factor with a potential impact on anorexia and reduced olfactory or gustatory performance. This has also been difficult or inconsistent in previous studies [30]. 

Moreover, longitudinal testing enabled monitoring CIOGD post-therapy. We found that olfactory and gustatory functions commenced to recover after completion of treatment. However, baseline levels for both olfaction and gustation were not attained within 6 to 12 months. Previous data on the duration of recovery are heterogeneous. Some describe full recovery within weeks to 3 months [23,54], while others describe 6 or 12 months [55,56]. This variability in data is most likely due to different types of cancer as well as different chemotherapy regimens. Moreover, most studies on CIOGD have not monitored patients post-therapy, which leads to scarce and, thus, insufficient data on the process of recovery. Hence, our findings highlight the need for further investigation of recovery of gustatory and olfactory function after completion of chemotherapy. 

Besides testing overall gustatory function, we also distinguished between the sensation of sweet, sour, salty, and bitter taste. Before, during, and after chemotherapy, sweet taste was perceived most frequently. Interestingly, the longer chemotherapy was received, the fewer patients were able to detect salty taste. In contrast, the sensation of bitter taste showed the strongest recovery, which might be due to its evolutionary importance as an indicator of toxins in foods [57]. These findings were consistent with numerous studies, though current data are not unambiguous yet. However, other studies that also used psychophysical tests also described the highest sensitivity for sweet taste, the lowest sensitivity for sour taste, and the strongest reduction for salty taste [45,58]. Patients need to be informed about potential gustatory alterations to prevent the negative consequences of overusing sugar or salt [58]. There have also been reports of periodic dynamics of sensitivity during each chemotherapy cycle [23]. This could also lead to varying results between studies depending on differing study designs. 

The qualitative assessment of CIOGD was performed using questionnaires and was found in 10 to 50% of patients during chemotherapy. Most often, patients reported parosmia or parageusia. These findings are consistent with previous studies, which also described higher rates as well as severe consequences of qualitative dysfunctions of olfaction and gustation during chemotherapy [51,59]. 

The strengths of our study involved the use of both self-assessments and standardized psychophysical tests to examine both taste and smell function. In addition, longitudinal examinations over the course of 12 to 18 months enabled us to monitor changes in olfactory and gustatory function during and after receipt of chemotherapy. Comparing groups with tc and pc offered additional information on long-term CIOGD as well as on chemosensory recovery post-therapy. In addition, we used neurological examinations and nerve conduction studies to identify correlations between chemosensory and neurological dysfunctions. These results convinced us to launch a follow-up study among patients diagnosed with lymphomas using olfactory interventions to assess the application of prevention and treatment strategies for CIOGD in the future. 

However, our study also had several limitations. First, we conducted the vast majority of patient examinations at the hospital or the outpatient department. We connected these examinations with patients’ regular appointments for treatment or follow-up. This, however, led to a certain variance regarding the exact timing and interval of examinations. Second, multiple reasons caused patients to drop-out throughout the course of our study, the most frequent being changes in residence or continuation of treatment at a hospital closer to their residence. Drop-outs rose to over 30% after T3. Third, we assessed qualitative dysfunctions only by means of questionnaires due to a lack of psychophysical examination methods. Fourth, we performed this study during the global pandemic of COVID-19. Although active infections were listed as exclusion criteria for participation and associated dysosmia or dysgeusia were conscientiously inquired, potential effects or overlap of chemosensory dysfunction cannot be ruled out entirely. Fifth, long-term chemotherapy regimens could be adjusted to better patients’ overall health and alleviate side effects. This might have led to altering levels of neurotoxicity, which, however, was only evaluated as a dichotomous trait and thus may not reflect each patient’s treatment in its entire complexity. Sixth, patients with various types of cancer were included in our study. This also led to greater heterogeneity in chemotherapy regimens, which did not allow for more precise analyses regarding correlations of CIOGD with particular types of cancer or chemotherapy regimens. Therefore, future research should focus on more homogeneous study cohorts of appropriate size in order to identify additional risk factors, such as type of cancer and chemotherapeutic substances with a high risk of CIOGD more accurately. 

## 5. Conclusions

In sum, this study extends prior research on CIOGD in adult cancer patients. Receipt of chemotherapy led to high rates of dysosmia and dysgeusia (90% and 78% of patients, respectively). We identified age (especially above 60 years), male sex, as well as reported anorexia as important risk factors. By means of nerve conduction studies, we identified correlations between CIOGD and symptoms of peripheral neuropathy. These findings suggest that CIOGD might be a neurotoxic effect of chemotherapy, which could lead to new therapeutic prospects. Furthermore, olfactory and gustatory recovery commenced post-therapy; however, full restoration was not attained within 6 to 12 months. This long-term impairment can significantly reduce patients’ quality of life and, therefore, requires future research to facilitate effective prevention and treatment strategies. Building upon our research results, we have launched the interventional “ImproverTox” trial to gain further insight and offer more support for patients suffering from CIOGD. 

## Figures and Tables

**Table 1:** Patient characteristics.

Parameter	All Patients	Patients Receiving tc	Patients Receiving pc
n	108	58	50
Mean age (SD) [years]	53.5 (17.6)	49.1 (19.1)	58.6 (14.1)
Female sex: n (%)	49.0 (45.4%)	22.0 (37.9%)	27.0 (54.0%)
Mean BMI (SD) [kg/m^2^]	24.7 (4.4)	24.2 (3.9)	25.2 (5.0)
Reported anorexia: n (%)	23.0 (21.3%)	16.0 (27.6%)	7.0 (14.0%)
ECOG-Score of 0: n (%)	101.0 (93.5%)	56.0 (96.6%)	45.0 (90.0%)
NT-treatment: n (%)	88 (81.5%)	48 (82.8%)	40 (80.0%)
Entity: n (%)			
Lymphoma	49 (45.4%)	46 (79.3%)	3 (6.0%)
Leukemia	13 (12%)	3 (5.2%)	10 (20.0%)
Gastrointestinal	46 (42.6%)	9 (15.5%)	37 (74.0%)

ECOG: Eastern Cooperative Oncology Group; NT: neurotoxic.

**Table 2:** Number of participating patients at each examination.

Examination	All Patients	Patients Receiving tc	Patients Receiving pc
T1	108	58	50
T2	91	52	39
T3	82	49	33
T4	63	39	24
T5	44	27	17

**Table 3:** Chemotherapy regimen applied to patients receiving tc in absolute numbers.

Regimen	T1	T2	T3	T4	T5
BEACOPP	14	13	11	10	7
Rituximab + Bendamustine	7	5	4	3	2
Rituximab + CHOP	19	18	18	16	14
GMALL (lymphoma)	4	4	4	3	3
FLOT	1	1	1	1	1
FOLFOX	3	3	3	2	2
Cisplatin + Etoposid	3	2	2	0	0
mFOLFIRINOX	1	1	1	1	1

**Table 4:** Chemotherapy regimen applied to patients receiving pc in absolute numbers.

Regimen	T1	T2	T3	T4	T5
GMALL (leukemia)	5	5	5	5	4
Doxorubicin + Cytarabine	9	9	8	5	2
Gemcitabine + Nab-paclitaxel	3	3	3	3	1
Gemcitabine + Cisplatin	4	4	3	3	1
FOLFOX	9	7	5	4	3
mFOLFIRINOX	15	10	9	5	4
VCD	2	2	2	2	2

## Data Availability

The raw data supporting the conclusions of this article will be made available by the authors upon request.
